# Advances in Machine Learning Paradigms for Reliable Swimming Pool Drowning Detection: Systematic Review of Methods and Implementations

**DOI:** 10.2196/100939

**Published:** 2026-11-03

**Authors:** Mercy Chepkorir, Idah Orowe, Elisha Abade, Timothy Kamanu

**Affiliations:** 1 Department of Mathematics, University of Nairobi, P.O Box 30197, Nairobi, 00100, Kenya, 254 0700590477; 2 Department of Computer Science, University of Nairobi, Nairobi, Kenya

**Keywords:** drowning detection, machine learning, computer vision, deep learning, real-time detection, multimodal sensing, evaluation metrics, privacy, water safety

## Abstract

**Background:**

Drowning is a leading cause of unintentional injury-related mortality worldwide, particularly in swimming pool environments, where rapid detection is critical to prevent fatalities. Traditional monitoring approaches rely on lifeguards and rule-based systems, which are often limited by human error, delayed response, and environmental challenges such as occlusion, glare, and multiple swimmers. Machine learning (ML) and computer vision methods have emerged as promising alternatives for automated drowning detection; however, their methodological rigor, real-world applicability, and reliability remain unclear.

**Objective:**

This study aimed to systematically review advancements in ML-based drowning detection systems over the past 15 years. The review focused on detection methods, sensing modalities, evaluation metrics, and validation practices. It also examined how studies addressed real-world challenges such as occlusion, lighting variability, and multiswimmer environments, and identified key gaps related to generalizability, reliability, and privacy.

**Methods:**

A systematic literature review was conducted using major electronic databases, including IEEE Xplore, Scopus, PubMed, Web of Science, ACM Digital Library, and Google Scholar. A total of 4927 records were initially identified and screened by 2 independent reviewers using predefined inclusion and exclusion criteria. A final set of 30 studies was included for data extraction and synthesis. The methodological quality and risk of bias were assessed using the Prediction Model Risk of Bias Assessment Tool (PROBAST), while reporting transparency was evaluated using the TRIPOD (Transparent Reporting of a Multivariable Prediction Model for Individual Prognosis or Diagnosis) guidelines. Data analysis was conducted in R.

**Results:**

The findings indicate that drowning detection research is dominated by supervised, vision-based approaches (23/30, 76.7%), with convolutional neural networks (5/30, 16.7%) and You Only Look Once–based architectures (7/30, 23.3%) being the most commonly used methods. While reported performance is generally high, with mean accuracy exceeding 90.9%, evaluation practices are heavily centered on accuracy, with limited reporting of precision, recall, false alarm rate, and latency. One of 30 (3.3%) studies reported validation under real-world operational conditions, with an additional 5 (16.7%) studies using real-world data without formal real-world validation, and no studies conducted cross-dataset evaluation. In addition, critical real-world challenges such as occlusion, glare, and crowding were rarely addressed (<10% of studies), and all studies relied on private datasets. The PROBAST assessment further indicated a high risk of bias in 96.7% (29/30) of studies, primarily due to limited validation and nonrepresentative data sources.

**Conclusions:**

ML-based drowning detection systems have demonstrated substantial progress in algorithm development, particularly in vision-based models. However, this progress is not matched by equivalent advances in validation, robustness, and real-world applicability. The field is characterized by a significant gap between experimental performance and deployment readiness. Future research should prioritize standardized evaluation frameworks, external validation, comprehensive performance reporting, and the integration of multimodal and privacy-preserving approaches to enable reliable real-world implementation.

## Introduction

Drowning remains a major global public health concern and a leading cause of unintentional injury-related mortality worldwide. Drowning is a major but preventable public health problem worldwide [1]. Each year, about 300,000 people die from unintentional drowning [2]. It is a leading cause of injury-related deaths. Young people are most affected. Over half of victims are under 25 years. Children aged 1 to 4 years are at the highest risk [3]. Most deaths, about 90%, occur in low- and middle-income countries due to limited safety measures and supervision.

Drowning is difficult to prevent because it is rapid and often silent, and victims show the instinctive drowning response. This includes vertical positioning and little movement. They are often unable to call for help. The time to intervene is very short, often seconds to minutes. Lifeguards play an important role but face limitations. Their performance is affected by fatigue, attention limits, and crowded environments [4]. Occlusion and high swimmer-to-lifeguard ratios make detection harder. These challenges highlight the need for automated, real-time monitoring systems to support human supervision.

Early technological approaches relied on rule-based systems and handcrafted features derived from visual observations. These methods attempted to detect distress through predefined patterns such as vertical body positioning and irregular motion. However, they lacked robustness and performed poorly in dynamic and real-world environments.

The emergence of machine learning (ML) and computer vision significantly advanced drowning detection research [5]. Convolutional neural networks (CNNs) were widely used for feature extraction from visual data [6]. Object detection models such as YOLO (You Only Look Once) and region-based approaches like Faster region-based CNN (R-CNN) improved real-time detection capabilities [7-10]. Recurrent neural networks and long short-term memory networks were applied to capture temporal patterns in swimmer behavior [11]. More recent studies explored spatiotemporal architectures and transformer-based models to model complex motion dynamics. Hybrid approaches combining multiple models further improved detection performance.

Despite substantial methodological progress, the field lacks a comprehensive and rigorous synthesis of existing evidence. Current studies are highly heterogeneous, with inconsistent reporting of evaluation metrics, limited use of operational performance indicators such as false alarm rates and latency, and minimal external validation. Most systems are evaluated in controlled environments using simulated scenarios, with little evidence of real-world deployment or cross-dataset generalization. These limitations make it difficult to assess the true effectiveness and readiness of existing systems for practical use.

This study therefore aimed to systematically review advancements in ML-based drowning detection methods published over the past 15 years. This time period was selected to capture the period during which modern ML, particularly deep learning and computer vision, emerged and rapidly evolved. The review examined detection models, data modalities, evaluation metrics, and deployment strategies. It also assessed how studies addressed real-world challenges and evaluated their methodological rigor.

Building on this trajectory, the remainder of this section traces the field’s technical evolution in greater depth. Automated drowning detection has evolved from heuristic observation models to data-driven ML systems. Early approaches identified drowning based on observable behavioral cues such as vertical body posture, limited forward motion, and irregular limb activity [12]. While these rule-based systems provided initial insights into drowning behavior, they were constrained by predefined thresholds and lacked robustness to environmental variability, including reflections, occlusion, and crowding. The introduction of classical ML methods improved automation by leveraging engineered features derived from motion and appearance. However, these approaches remained dependent on feature design and exhibited limited generalizability in complex aquatic environments [5].

The adoption of deep learning marked a significant shift in the field, enabling end-to-end learning of spatial and temporal representations directly from visual data. CNNs became widely used for feature extraction, while object detection frameworks such as YOLO have been extensively applied for real-time swimmer localization and monitoring [13,14]. More advanced architectures, including feature pyramid networks, Mask R-CNN, and pose estimation models, have further improved detection performance under varying conditions [11]. Despite these advances, most systems rely on supervised learning, which requires labeled datasets that are scarce for rare events such as drowning [11,15]. Research in this domain remains dominated by frame-based detection approaches, with comparatively limited exploration of temporal modeling or anomaly detection strategies.

Recent work has also explored alternative sensing modalities, including underwater cameras, wearable devices, radio-frequency identification (RFID) systems, and depth-based approaches. While these modalities aim to improve robustness under challenging conditions, vision-based systems remain dominant due to their practicality and scalability [16]. Across the literature, key challenges persist, including sensitivity to environmental factors such as glare and occlusion, limited validation in real-world settings, and the absence of standardized datasets. These limitations highlight the need for a systematic synthesis of existing approaches, particularly in relation to methodological trends, evaluation practices, and deployment readiness. The evolution practices have been illustrated in Figure 1.

**Figure 1.**
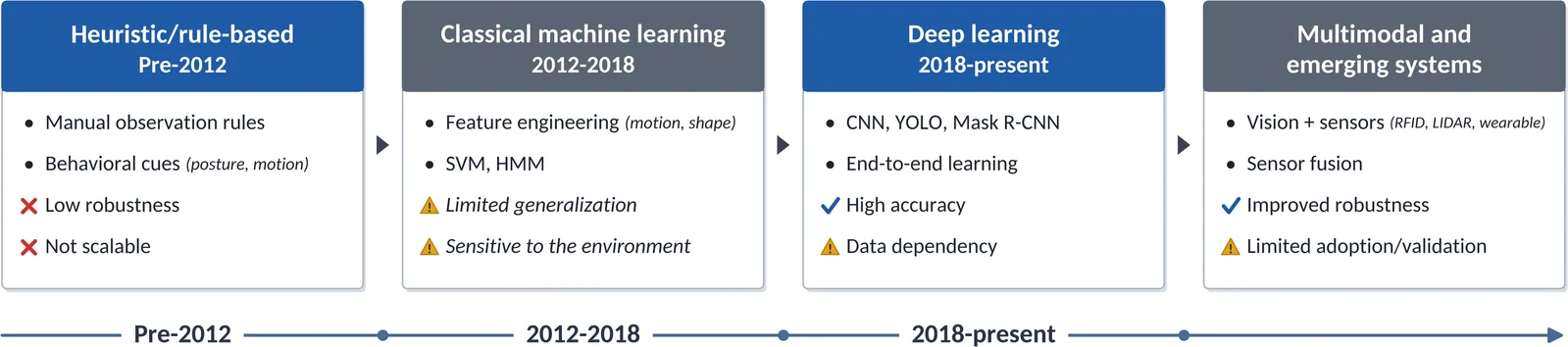
Evolution of drowning detection. CNN: convolutional neural network; HMM: hidden Markov model; LiDAR: light detection and ranging; Mask R-CNN: mask region-based convolutional neural network; RFID: radio-frequency identification; SVM: support vector machine; YOLO: You Only Look Once.

## Methods

### Goal and Review Questions

#### Goal of the Study

The main goal of this study was to systematically map, review, and synthesize studies that focused on ML-based drowning detection. This included an in-depth examination of detection models, data modalities, deployment strategies, and practical applications of these systems. The study also examined how different approaches addressed real-world challenges such as occlusion, glare, and the presence of multiple swimmers.

This study aimed to identify recent trends, coverage, and directions in drowning detection research. It also aimed to highlight key limitations related to generalizability, reliability, and privacy. These insights were intended to inform future research and support the development of robust and deployable detection systems. The protocol was defined before data collection, thereby minimizing the risk of bias in study selection and reporting. The protocol has been registered with the Open Science Framework before data collection. Details of the registered protocol may be found in [17]. This ensured absolute transparency, reproducibility, and minimized risk of bias in study selection and reporting.

Based on established guidelines for conducting systematic literature reviews and reporting findings, the selection and review process was carefully designed and documented. Based on this goal, the following review questions (RQs) were formulated and adopted.

#### Category 1: General RQs for the Systematic Review

The following research questions were general to all systematic literature review studies:

1. RQ 1.1. Contributions presented in the studies: What were the main contributions of the reviewed studies? This question focused on identifying whether the studies contributed through the development of models, algorithms, systems, datasets, or practical applications for drowning detection.
2. RQ 1.2. Research methods used in the studies: How were the studies categorized based on research methods? This question aimed to classify the studies based on whether they employed empirical, experimental, or hybrid approaches, given the multidisciplinary nature of drowning detection systems.

#### Category 2: RQs Specific to the Topic of Systematic Review

The following RQs were specific to the topic of advances in ML-based drowning detection:

1. RQ 2.1. Methods and models employed for drowning detection: What methods and models were employed for detecting drowning incidents? This question aimed to identify whether studies used classical approaches, ML models, deep learning architectures, or hybrid methods. It also examined the use of specific models such as CNNs, object detection frameworks, pose estimation, and spatiotemporal models.
2. RQ 2.2. Data modalities and feature representation: What types of data and feature representations were used in the studies? This question focused on whether studies used vision-based data, sensor-based data, or multimodal approaches. It also examined how spatial, temporal, and motion-related features were represented and used for detection.
3. RQ 2.3. Types and sources of datasets: What datasets were used in the studies? Were they primary datasets collected by researchers or secondary datasets obtained from existing sources?
4. RQ 2.4. Evaluation metrics and reported performance: What evaluation metrics and performance measures were reported? This question examined metrics such as accuracy, precision, recall, *F*_1_-score, false alarm rate, and latency. It aimed to compare the effectiveness of different models and assess consistency in reporting.
5. RQ 2.5. Real-time capability and deployment: Were the proposed models capable of real-time detection, and were they deployed in practical settings? This question evaluated whether systems were implemented in real-world environments such as swimming pools, surveillance systems, or embedded devices, and how they handled operational constraints.
6. RQ 2.6. Handling of real-world challenges: How did the studies address real-world challenges such as occlusion, glare, lighting variability, and multiple swimmers? This question examined whether the models were robust under complex and dynamic conditions.
7. RQ 2.7. Generalizability and external validation: To what extent did the studies evaluate generalizability? This question assessed whether models were tested across different datasets, environments, or populations, and whether external validation was performed.
8. RQ 2.8. Privacy and data security considerations: How did the studies address privacy and data security? This question examined whether privacy-preserving approaches, such as federated learning, were used or discussed.
9. RQ 2.9. Limitations and risk of bias: What limitations and risks of bias were identified in the studies? This question focused on issues related to data quality, study design, lack of validation, and incomplete reporting, as observed in the risk of bias assessment.

### Search Strategy

To ensure transparency and completeness in our systematic review, we followed PRISMA (Preferred Reporting Items for Systematic Reviews and Meta-Analyses) guidelines for conducting systematic literature reviews as outlined by relevant studies. Our search spanned 6 databases, covering literature from 2012 to 2025, with the aim of identifying relevant articles on the methodologies, deployment strategies, and practical applications in drowning detection. Records published between 2012 and 2018 were identified during the search but did not meet the study’s inclusion criteria at the full-text screening stage, primarily because they described rule-based or handcrafted-feature detection approaches rather than ML-based systems, and were therefore excluded under the exclusion criterion. All 30 included studies fall within the 2019 to 2025 period, consistent with the rapid emergence of deep learning–based approaches in this field during that time.

The databases used for this review include IEEE Xplore, Web of Science Core Collection, Scopus, PubMed, ACM Digital Library, and Google Scholar. The databases and full search terms are provided in Multimedia Appendix 1. The search strings were developed and structured using the PICO framework, which considers the Problem, Intervention, Comparison, and Outcome components (Multimedia Appendix 2) [18]. It was designed to address the overarching question: “Can advanced ML methodologies enhance drowning detection and improve real-time response in water environments?” We tailored the PICO structure as follows:

1. Comparison: Due to the specificity of our research objectives, direct comparison studies were not applicable in this context.
2. Outcome: We sought outcomes related to accuracy, deployment, and practical applications of drowning detection methodologies, incorporating terms such as “accuracy” OR “precision” OR “recall” OR “*F*_1_-score” OR “false alarm rate” OR “real-time” OR “deployment” OR “latency.”

By employing “AND” and “OR” operators, we formulated a general search phrase:

(“drowning detection” OR “drowning recognition” OR “swimmer distress” OR “near-drowning” OR “water safety monitoring”) AND (“machine learning” OR “deep learning” OR “computer vision” OR “neural networks” OR “YOLO” OR “CNN” OR “LSTM” OR “pose estimation”) AND (“accuracy” OR “precision” OR “recall” OR “*F*_1_-score” OR “false alarm rate” OR “real-time” OR “deployment” OR “latency”)

### Eligibility Criteria

The eligibility criteria for inclusion were established to comprehensively evaluate studies that focused on ML-based drowning detection. The criteria considered a wide range of methodologies, data modalities, and application contexts. They were carefully designed to ensure the inclusion of studies aligned with the objectives of this review.

The studies were evaluated to determine whether they addressed key aspects of drowning detection, including detection methods, deployment strategies, real-time capabilities, and practical applications. Emphasis was placed on studies that examined challenges such as occlusion, lighting variability, and multiswimmer environments. The criteria also ensured the inclusion of studies that contributed to understanding trends, limitations, and future research directions in drowning detection.

### Initial Screening

During this preliminary phase, articles identified through database searches and manual exploration underwent early screening based on titles and abstracts. The aim was to ensure that the screened studies aligned with the goal of systematically reviewing ML-based drowning detection.

Emphasis was placed on filtering out irrelevant studies and identifying those contributing to the analysis of detection methods, data modalities, deployment strategies, performance evaluation, and practical applications. Studies not related to drowning detection or not involving computational or ML approaches were excluded at this stage.

Adherence to established systematic review practices ensured comprehensive documentation of the selection process. This supported the formulation of the research questions and the synthesis of findings.

### Inclusion and Exclusion

Out of 4927 search results, a total of 30 studies were included for review. Studies that were included had to fulfill the following criteria: (1) studies that focused on ML-based drowning or swimmer distress detection, (2) studies that detailed the methodologies or models used for detection, and (3) studies that clearly defined evaluation metrics, deployment strategies, or practical applications of drowning detection techniques.

To ensure precise and robust findings, this study used exclusion criteria guided by specific thematic considerations. (1) Irrelevant topics: literature investigating other aspects of aquatic monitoring not related to drowning or distress detection. (2) Incomplete focus: studies focused solely on other processing tasks, such as preprocessing or noise reduction, without subsequent detection or classification. (3) Lack of technological interventions: literature that did not apply ML, computer vision, or related computational approaches, such as studies focusing purely on theoretical or descriptive aspects without implemented detection systems. (4) Insufficient application: literature that only described swimmer behavior, features, or observations without using them in detection models. (5) Limited scope: studies that focused solely on general surveillance, swimmer tracking, or water safety without addressing drowning or distress detection. (6) Non-English publications: studies that were not available in English due to language proficiency constraints of the review team.

A 2-stage screening process was used to ensure thoroughness and reduce the bias arising. Two reviewers independently screened all titles and the corresponding abstracts in the first step to assess relevance based on the inclusion criteria. Afterward, the papers were advanced to the next stage if and when at least 1 reviewer deemed them relevant. In cases where consensus at the title and abstract level could not be achieved between the 2 reviewers, an adjudication by a third person was sought on a consultation basis and on the purpose. During the second stage, 2 independent reviewers performed a full-text blind review. Consensus was obtained through deliberation between reviewers when required. Consensus meetings were held to resolve any disagreements and uncertainties. The objectivity of the criteria was assessed through reviewer agreement during the screening stages. The study selection process is presented in Figure 1.

### Data Synthesis and Comprehensive Analytical Approach

In alignment with the RQs, the following information points were recorded and analyzed: (1) year of publication, (2) contribution of each study, (3) categorization of each study based on research approach, (4) ML methods and model architectures, (5) data modalities employed (eg, red, green, and blue [RGB] video, underwater video, sensor-based, or multimodal data), (6) deployment characteristics including real-time capability and hardware requirements, (7) practical application settings (eg, swimming pools, open water environments, surveillance systems), (8) reported performance metrics such as accuracy, precision, recall, *F*_1_-score, false alarm rate, and latency, and (9) challenges addressed, including occlusion, lighting variability, multiswimmer environments, and viewpoint variation.

Tables and graphical summaries were developed to synthesize these information points, providing a comprehensive overview of advancements in drowning detection methods, system design, and real-world applicability. The temporal distribution of publications indicated a growing research interest in recent years, reflecting the increasing importance of automated drowning detection systems and the advancement of ML techniques in safety-critical environments. Meta-analysis was not undertaken because the included studies were heterogeneous in model, evaluation metrics reported, dataset type, and study design. This degree of methodological and outcome heterogeneity precluded meaningful statistical pooling, consistent with PRISMA guidance favoring narrative and tabular synthesis under such conditions [19].

### Quality Assessment

The objective of this quality assessment was to evaluate the significance and validity of each selected document in the context of ML-based drowning detection. Although the quality rating did not significantly influence the selection of the primary studies, the assessment aimed to reflect the overall validity and reliability of the included studies.

The TRIPOD (Transparent Reporting of a Multivariable Prediction Model for Individual Prognosis or Diagnosis) [20] standard was employed as a measure of reporting quality. Each article was scored based on adherence to the TRIPOD checklist, with binary scores assigned as either 1 (compliant) or 0 (noncompliant). Moreover, all reviewed studies were expected to meet a minimum threshold of reporting quality to ensure clarity and reproducibility. A detailed checklist of the TRIPOD assessment is provided in .

The TRIPOD checklist was used to evaluate the reporting clarity and comprehensiveness across all selected studies. Each study was assessed based on key TRIPOD components, including data description, model development, validation procedures, and reporting of results. The assessment highlighted variability in reporting practices across studies, particularly in relation to external validation, dataset transparency, and performance metric reporting. Overall, the application of TRIPOD ensured a structured evaluation of reporting quality and supported the reproducibility of the findings.

The study additionally employed the PROBAST (Prediction Model Risk of Bias Assessment Tool) to assess the risk of bias in the included studies [20]. Each study was scored across 5 core TRIPOD reporting domains: Title/Abstract, Introduction, Methods, Results, and Discussion, rather than scoring each of the checklist’s individual items separately. Each domain was scored as 1 (adequately reported) or 0 (not adequately reported), yielding a total domain-level score out of 5 per study; this approach was adopted to provide a tractable, reproducible summary measure of reporting quality across a heterogeneous set of study designs, while a detailed item-level checklist is provided in Checklist 1 [21]*.* The PROBAST framework was considered appropriate given the predictive modeling nature of drowning detection systems. It is acknowledged that PROBAST and TRIPOD were originally developed for clinical multivariable prediction models used for individual prognosis or diagnosis, and that applying them to computer vision object detectors, including YOLO variants, Mask R-CNN, and wearable sensor systems, represents an extension beyond their original scope. This extension was considered justified because the included studies, despite differing in architecture, share the core structure of a prediction model. They take input data such as images, video frames, or sensor signals and output a binary or probabilistic prediction (drowning vs nondrowning), which is the fundamental unit of analysis PROBAST and TRIPOD were designed to appraise. Domain-specific tools for AI/ML diagnostic studies, such as Quality Assessment of Diagnostic Accuracy Studies 2 (QUADAS-2), Checklist for Artificial Intelligence in Medical Imaging (CLAIM), and CLEAR, were considered as alternatives; however, PROBAST and TRIPOD were retained because they offer the most established, validated domain structure for systematically assessing risk of bias and reporting transparency across heterogeneous prediction-model designs, and because comparable systematic reviews in adjacent safety-critical ML domains have applied the same tools. The risk of bias assessment covered 4 key domains: (1) bias related to participants and datasets, (2) bias in predictor selection and feature representation, (3) bias in outcome definition and measurement, and (4) bias in model development and analysis.

The results of the quality and risk of bias assessments were summarized in Table 1, with detailed individual evaluations provided in Multimedia Appendix 3.

**Table 1.** Risk of bias assessment (N=30).

Domain	Low risk, n (%)	Some concerns, n (%)	High risk, n (%)
Data source bias	4 (13.3)	10 (33.3)	16 (53.3)
Prediction bias	27 (90)	3 (10)	0 (0.0)
Outcome assessment bias	1 (3.3)	28 (93.3)	1 (3.3)
Analysis bias	11 (36.7)	10 (33.3)	9 (30)
Overall risk	0 (0.0)	1 (3.3)	29 (96.7)

The assessment of data source bias indicated that a substantial proportion of studies, 53.3% (16/30)**,** were considered to have a high risk of bias, largely due to reliance on limited, nonrepresentative, or synthetic datasets. A further 33.3% (10/30) of studies raised some concerns, while only 13.3% (4/30) were assessed as low risk. These findings suggest that data-related limitations remain a major challenge, potentially affecting the generalizability and robustness of the reported results.

The assessment of prediction bias showed that 90% (27/30) of studies had a low risk of bias, indicating that most studies employed appropriate modeling techniques and prediction strategies. Only 10% (3/30) of studies presented some concerns, suggesting that predictive modeling approaches were generally well implemented across the reviewed literature.

The assessment of outcome assessment bias revealed a significant area of concern, with 93.3% (28/30) of studies classified as having some concerns, primarily due to inconsistencies in evaluation protocols and lack of standardized performance metrics. Only 3.3% (1/30) of studies were considered low risk, while 3.3% (1/30) were categorized as high risk. These findings indicate that outcome evaluation remains insufficiently standardized, limiting comparability across studies.

The assessment of analysis bias indicated a relatively balanced distribution, with 36.7% (11/30) of studies categorized as low risk, 33.3% (10/30) showing some concerns, and 30% (9/30) identified as high risk. This variability reflects differences in validation strategies, with several studies lacking external validation or relying on limited experimental setups, which may introduce overfitting and reduce reliability.

The overall assessment of risk of bias indicated that 96.7% (29/30) of the studies posed a high risk, while only 3.3% (1/30) showed some concerns. No studies were classified as low overall risk. These findings highlight substantial methodological limitations across the literature, particularly related to dataset quality, evaluation consistency, and real-world validation.

The TRIPOD assessment indicates generally strong reporting practices, with an average score of 4.07 (81%) out of a maximum of 5 (one point per reporting domain: Title/Abstract, Introduction, Methods, Results, and Discussion). Domain-level compliance varied substantially: Title/Abstract and Introduction were adequately reported in 100% (30/30) of studies, Discussion in 86.7% (26/30), Results in 70.0% (21/30), and Methods in only 50% (15/30), the weakest-performing domain, consistent with this review’s broader finding of limited methodological transparency. Overall**,** 24 studies were classified as having high reporting quality (domain score of 4/5 or 5/5), 5 studies as having moderate reporting quality (3/5), and 1 study as having low reporting quality (2/5 or below). This suggests that most studies provided clear descriptions of model development and reported performance metrics adequately. However, important gaps remained, particularly in relation to external validation, dataset transparency, and reproducibility details. These limitations affect the interpretability and generalizability of the findings despite strong reporting compliance.

The combined risk of bias and reporting quality assessments indicate that, while the field demonstrates strong methodological progress in model development and reporting clarity, there are significant gaps in data quality, evaluation standardization, and real-world validation.

## Results

The findings in this section are structured according to the classification of the reviewed studies and the key technical considerations relevant to contextualizing drowning detection systems (Figure 2).

**Figure 2.**
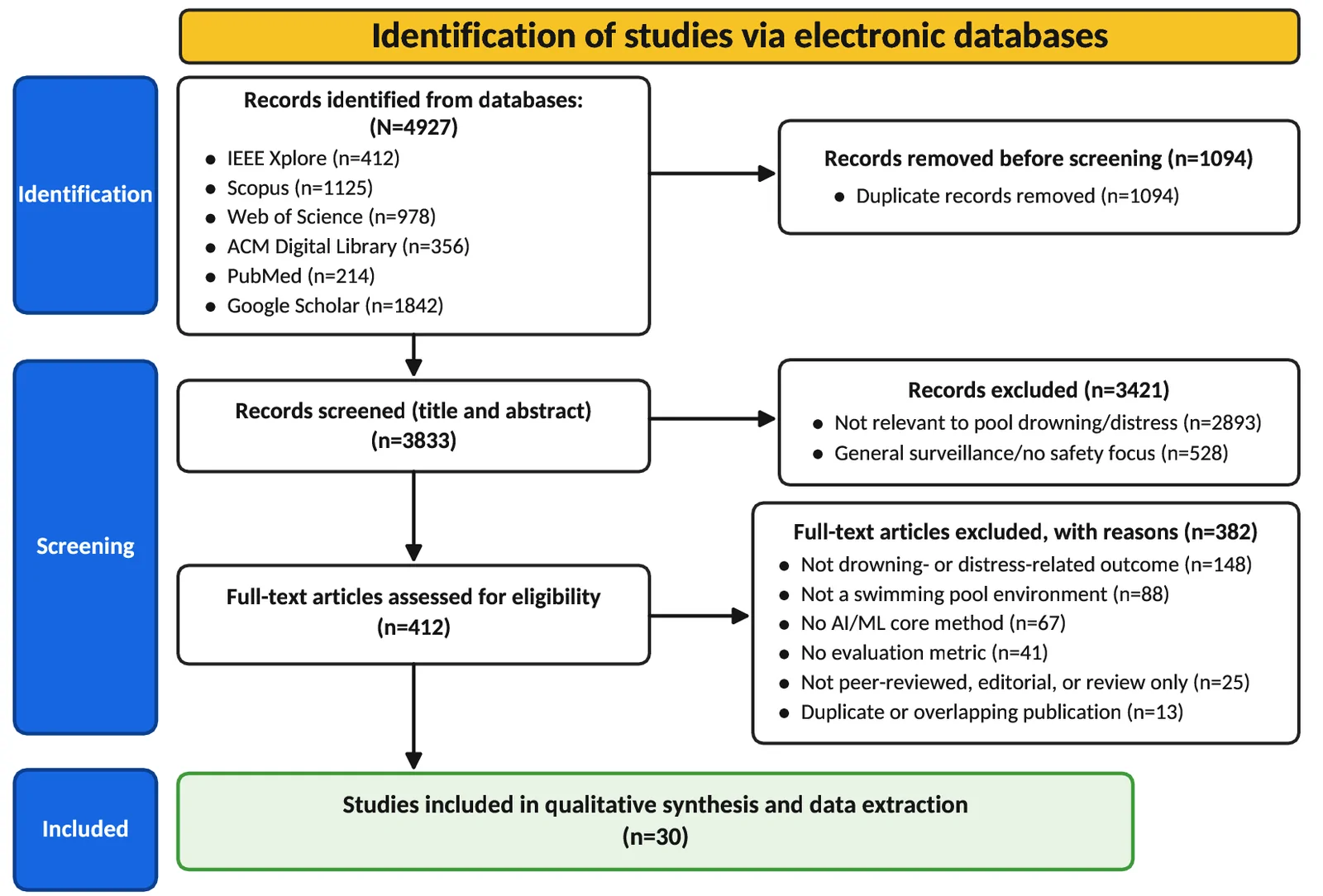
PRISMA (Preferred Reporting Items for Systematic Reviews and Meta-Analyses) flowchart. ML: machine learning.

### Overview of Included Studies

A total of 30 studies published between 2019 and 2025 were included in this review. The temporal distribution demonstrates a clear and rapid increase in research activity over time. Publication output remained minimal in the early years, with 3.3% (n=1) of studies published in both 2019 and 2020, followed by a sharp increase in subsequent years and peaking in 2024. This trend reflects the accelerating interest in ML-based drowning detection systems, particularly in recent years.

Geographically, research output was highly concentrated. China accounted for 36.7% (11/30) of the included studies, emerging as the dominant contributor. This was followed by the United Arab Emirates, Thailand, Australia, and India, each contributing 6.7% (2/30). Nine additional countries, Saudi Arabia, Canada, Spain, Sri Lanka, Pakistan, Taiwan, Russia, Peru, and the Philippines, each contributed a single study (3.3%). The dataset’s country of origin could not be determined for 3 (10%) studies, and one additional study was a China and Pakistan collaboration counted toward both countries, which is why the percentages sum to slightly more than 100%. As illustrated in Figure 3, the recent surge in publications is largely driven by contributions from China across 2023 to 2025.

**Figure 3.**
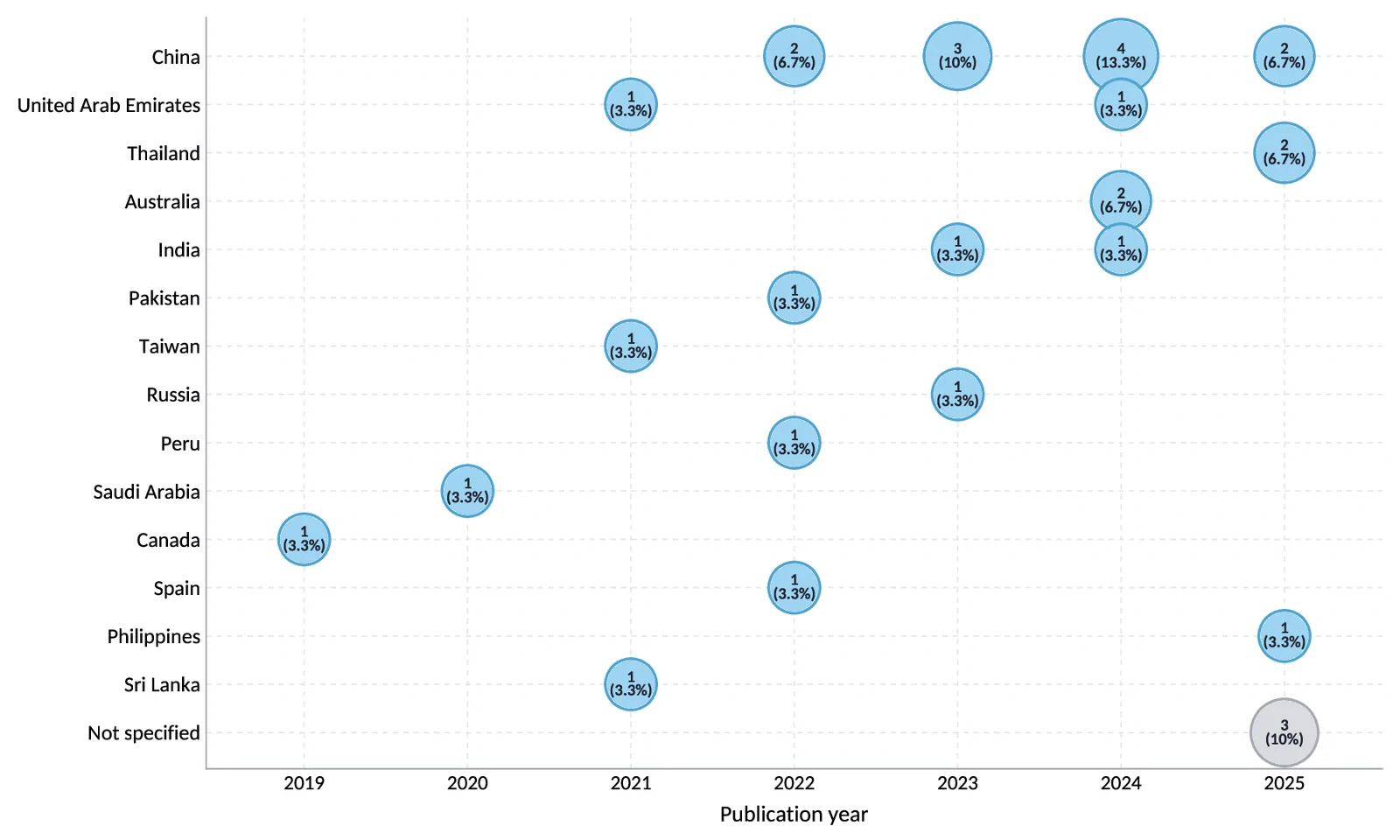
Distribution of included studies by country and publication year. Bubble size represents number of studies; labels show study count and percentage of total studies (N=30).

These findings indicate that while the field is rapidly expanding, it remains geographically concentrated. This concentration suggests that current advancements are shaped by regions with greater access to technological infrastructure and data resources. The diversity of environmental contexts represented in the literature remains limited, raising concerns about the generalizability and real-world applicability of existing drowning detection systems.

### Methodological Approaches

The methodological landscape of the included studies was heavily dominated by detection-oriented approaches, which accounted for 70% (21/30) of all studies. Systems combining detection with early warning mechanisms represented 10% (3/30)**,** while classification or recognition-based approaches constituted only 6.7% (2/30). Other methodological paradigms, including pose-based detection, anomaly-assisted detection, multimodal systems, and dataset-focused studies, were each represented by 3.3% (1/30) of the included studies. This distribution highlights a strong emphasis within the literature on direct event detection and real-time alert generation, reflecting the practical importance of timely intervention in drowning scenarios [10,22,23]. However, it also reveals a relative underrepresentation of approaches focused on behavioral modeling, temporal dynamics, and probabilistic inference**,** which are critical for capturing the progressive and often subtle nature of drowning events.

Despite the predominance of detection-based methods, most studies addressed only a limited subset of real-world operational challenges. In particular, key issues such as crowd density and multiswimmer interactions**,** as well as class imbalance arising from the rarity of true drowning events**,** were rarely incorporated into model design or evaluation frameworks [24]. Additionally, environmental distortions**,** including underwater visibility challenges, were predominantly handled in studies specifically tailored to underwater settings [15,25]. This suggests that robustness in current systems is often context-specific rather than generalizable across diverse aquatic environments.

### Learning Paradigms and Model Architecture

Supervised learning overwhelmingly dominated the methodological landscape, accounting for 96.7% (29/30) of the included studies. Only a single study (1/30, 3.3%) employed a semisupervised anomaly detection framework, highlighting the limited exploration of alternative learning paradigms such as unsupervised or self-supervised approaches [26].

In terms of model architecture, the YOLO family of object detection models emerged as the most frequently utilized, appearing in 23.3% (7/30) of studies. This included variants such as YOLOv5, YOLOv7, YOLOv8, and YOLOv5n, reflecting the field’s strong preference for real-time, detection-oriented pipelines. Generic CNN architectures were also widely used, accounting for 16.7% (5/30) of studies. Similarly, 16.7% (5/30) of studies reported the use of deep learning approaches without clearly specifying the underlying architecture, indicating a lack of transparency in model reporting.

Other architectures were less frequently represented and included Mask R-CNN, feature pyramid networks, transfer learning–based systems, neural network–based classifiers, YOLOv8-Pose, and YPMNet, each contributing 3.3% (1/30) of the total studies [7,27]. Notably, 20% (6/30) of studies did not provide sufficient detail regarding their model architecture.

### Sensing Modalities

RGB video was the most prevalent sensing modality, used either alone in 40% (12/30) of the included studies or in combination with one or more additional modalities in a further 30% (9/30) of studies (6 studies paired RGB video with either wearable/sensor data or underwater camera footage; 3 studies combined RGB video with two further modalities, underwater camera plus wearable sensing, sonar, or sonar and light detection and ranging).

Non-RGB sensing modalities were used in a further 20% (6/30) of studies: wearable or smartphone-based sensing alone (n=3), underwater camera alone (n=1), sonar alone (n=1), and underwater camera combined with sonar (n=1). The sensing modality could not be determined for the remaining 10% (n=3), as shown in Figure 4.

**Figure 4.**
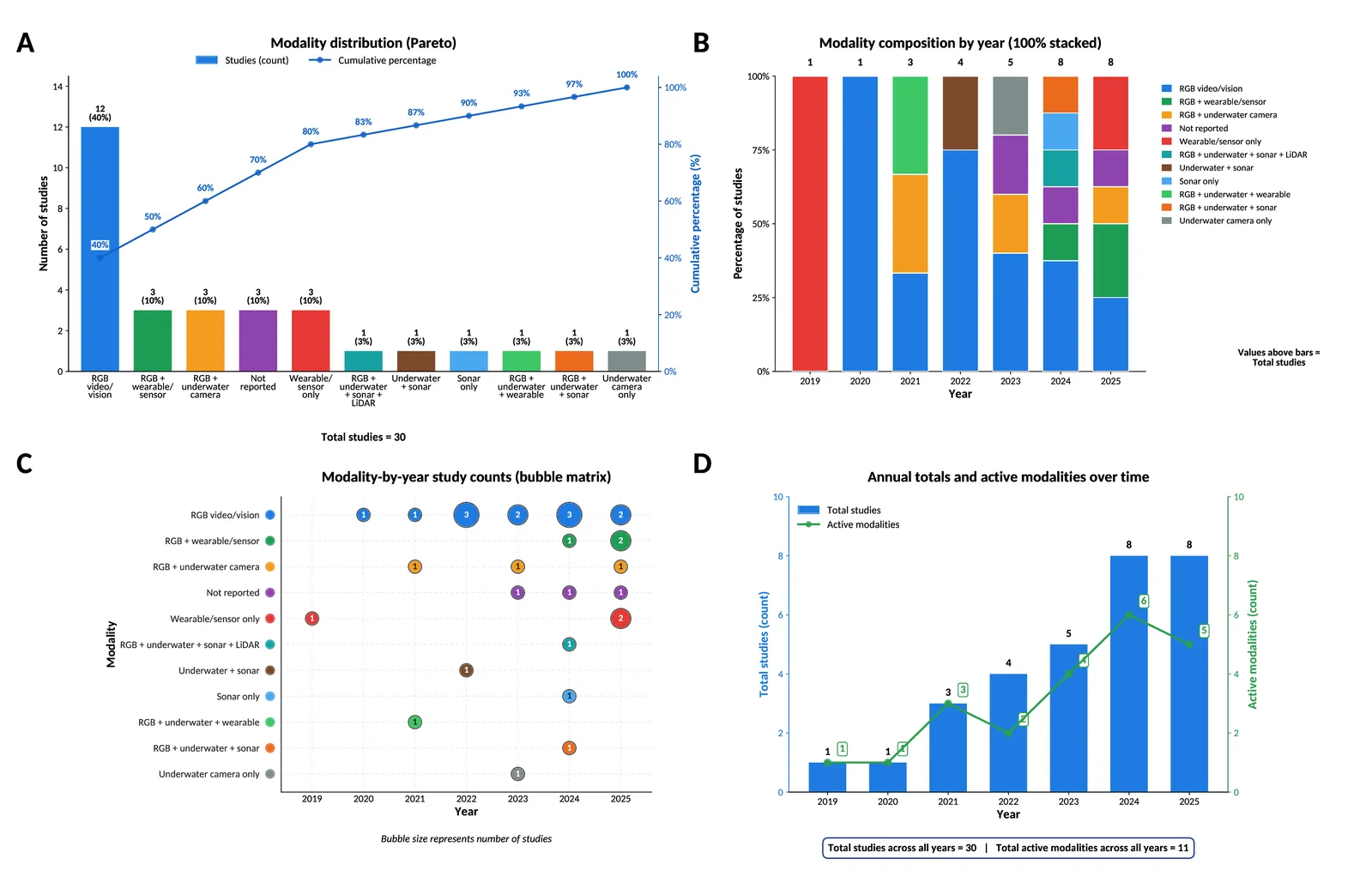
Distribution of sensing modality over time. LiDAR: light detection and ranging; RGB: red, green, and blue.

These findings indicate that the field is heavily dominated by vision-based modalities, particularly RGB and underwater imaging [13,28]. In contrast, multimodal and sensor-fusion approaches remain underexplored [29]. Notably, robustness to underwater distortion appears largely confined to systems explicitly designed for underwater environments, reinforcing the modality-specific nature of current solutions and highlighting a gap in generalizable, cross-modal detection frameworks [16].

### Performance Metrics

Accuracy was the most frequently reported evaluation metric, appearing in 56.7% (17/30) of the included studies. Among these studies, the mean reported accuracy was 90.9%, with a median of 90%, and values ranging from 72.17% to 100%. The highest reported accuracies included 100%, 99.4%, and 99%, indicating generally strong classification performance across studies.

Precision was reported in 36.7% (11/30) of studies, with values ranging from 73.91% to 98%**,** while recall was reported in 33.3% (10/30). In contrast, *F*_1_-score and related classification metric negative trade-offs were reported inconsistently, limiting direct comparability across studies as shown in Figure 5.

**Figure 5.**
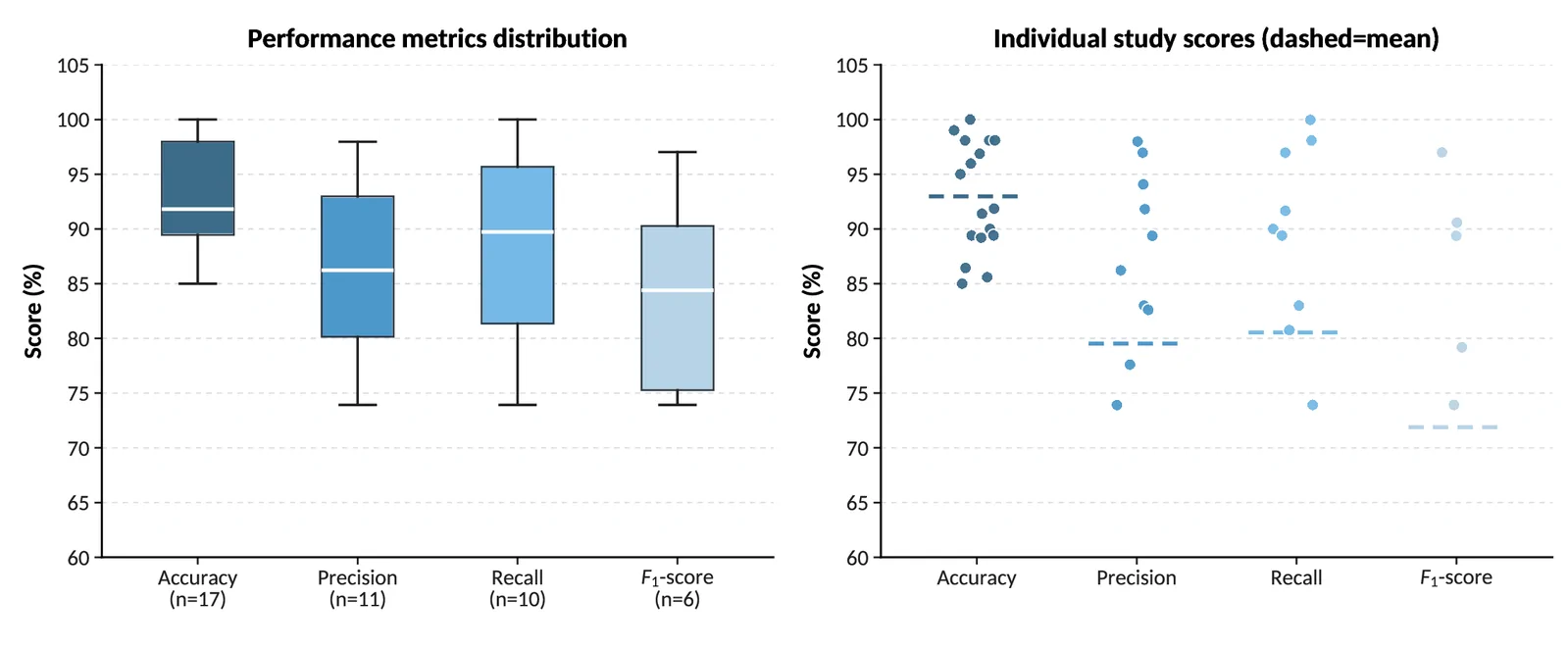
Reported performance metrics distribution.

Overall, the metric profile of the field is heavily centered on accuracy, despite drowning being a rare event relative to normal swimming activity. This imbalance raises concerns about the adequacy of accuracy as a standalone metric. The limited and inconsistent reporting of precision, recall, and *F*_1_-score constrains the ability to evaluate false positive and false negative trade-offs, which are critical factors for real-world deployment.

Operational performance metrics were notably scarce. Latency was reported in only 3.3% (1/30) of studies, while frame rate was reported in 20% (6/30), with values including 118.6 FPS, 31 FPS, 30 FPS, 22.9 FPS, and 5 FPS. This suggests that the literature remains largely focused on classification performance, with limited emphasis on real-time feasibility and deployment constraints.

### Real-Time Capability and Validation

A total of 46.7% (14/30) of the included studies reported real-time capability. However, only 3.3% (1/30) provided empirical validation demonstrating both real-time performance and evaluation under real-world operational conditions.

The remaining 43.3% (13/30) of studies claimed or implied real-time operation without providing sufficient validation evidence, while 53.3% (16/30) made no reference to real-time capability, as shown in Figure 6.

**Figure 6.**
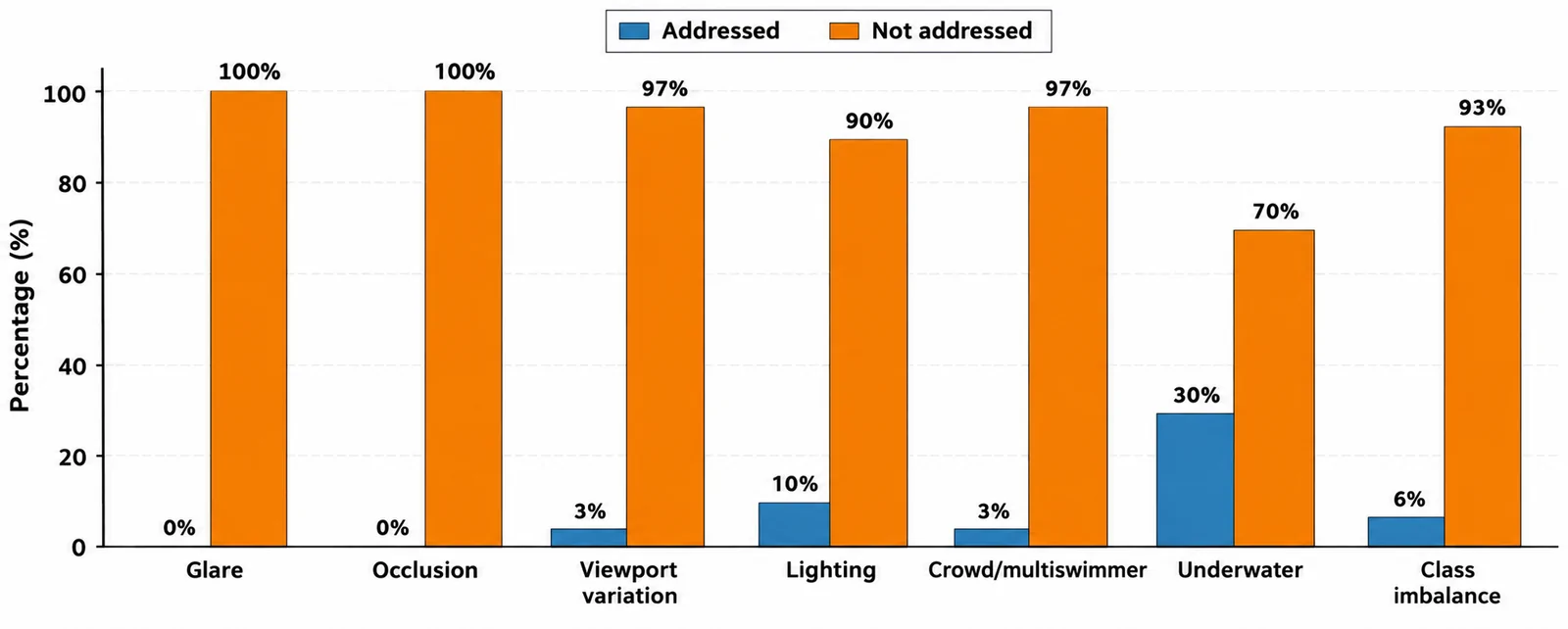
Real-world challenges addressed by studies.

This gap between reported capability and validated performance is further reflected in the limited reporting of operational metrics. Latency and frame rate (FPS) were inconsistently documented across studies, restricting the ability to assess whether proposed systems meet real-time deployment requirements. These findings indicate that while real-time detection is frequently presented as a core objective, it remains largely unverified in practice.

### Dataset Description and Reporting Practices

The reporting of dataset characteristics across the included studies was limited and inconsistent, raising concerns about transparency and reproducibility. A substantial majority of studies, 80% (24/30), did not clearly specify whether their datasets were real, simulated, or synthetic. Only 16.7% (5/30) explicitly reported the use of real-world data, while 3.3% (1/30) used simulated data.

This lack of clarity limits the ability to assess the ecological validity of reported performance outcomes and undermines confidence in how well these systems would generalize to real-world settings [25].

In addition, all studies reviewed relied exclusively on private datasets, with no use of publicly available benchmark datasets. The absence of shared datasets restricts comparability across studies and significantly limits reproducibility, both of which are critical for advancing the field.

Sample size reporting was also inconsistent. A total of 43.3% (13/30) of studies did not report sample size. Among those that did, there was substantial variability, ranging from as few as 17 samples to as many as 8572 images, indicating a lack of standardization in dataset scale and evaluation practice (Table 2).

**Table 2.** Summary table.

Method category	Representative approaches	Key achievements	Key limitations (as reported in studies)
Object detection (YOLO^a^-based)	YOLOv5, YOLOv7, YOLOv8, YOLOv8-Pose [23,27,29-32]	Achieved high detection performance, for example, accuracy up to 98%, high mean average precision values, demonstrated suitability for real-time detection; strong object localization capabilities	Several studies explicitly reported reliance on self-collected datasets and lack of external validation; key limitations include absence of false alarm rate reporting, limited real-world evaluation, and generalizability concerns
CNN^b^-based	CNN, Mask R-CNN^c^, ResNet-based models [22,33-40]	Demonstrated strong classification performance (85%-96% accuracy); effective spatial feature learning; widely adopted for drowning recognition tasks	Studies reported lack of real-world deployment, small or simulated datasets, and absence of comprehensive evaluation metrics (eg, AUC^d^, latency); limitations also include restricted generalizability across environments
Pose-based detection	YOLOv8-Pose, pose estimation methods [31,41,42]	Improved representation of human posture and motion; enhanced interpretability of drowning-related behaviors	Reported limitations include sensitivity to occlusion, difficulty handling multiple swimmers, and lack of real-world testing scenarios; studies also note limited evaluation datasets
Underwater computer vision	Underwater CNNs, YOLO-based underwater models [16,42-44]	Achieved high accuracy in underwater datasets (up to ~96%); effective handling of underwater distortion and visibility conditions	Studies reported limited applicability outside underwater settings, lack of cross-environment validation, and restricted dataset diversity; also noted absence of real-time validation in practical settings
Temporal/spatiotemporal models	Temporal anomaly detection, motion-based models [25,33]	Capture temporal dynamics of drowning behavior; suitable for detecting abnormal motion patterns over time	Reported limitations include computational complexity, lack of standardized evaluation metrics, and limited validation on real-world continuous video streams; also insufficient reporting of latency and deployment feasibility
Multimodal systems	Vision+LiDAR^e^/multimodal fusion [29]	Improved robustness through sensor fusion; enhanced contextual understanding; potential to reduce false positives	Study explicitly reported system complexity, lack of large-scale evaluation, and absence of real-world deployment validation; also noted limited benchmarking against standard vision-based approaches
Sensor-based/wearable systems	RFID^f^, wearable devices, embedded sensing [33,41,45-48]	Robust to visual challenges such as glare and occlusion; enables continuous monitoring independent of camera systems	Studies reported very small sample sizes, limited scalability, and dependency on user compliance; also lack of comprehensive performance evaluation metrics and absence of real-world deployment validation
Anomaly detection/semisupervised learning	Semisupervised and anomaly-based models [13,49]	Suitable for rare-event detection; reduces reliance on labeled drowning data; captures deviations from normal swimming patterns	Reported limitations include lack of interpretability, absence of standardized evaluation benchmarks, and limited comparative analysis with supervised methods; also minimal real-world validation

a YOLO: You Only Look Once.

b CNN: convolutional neural network.

c Mask R-CNN: mask region-based convolutional neural network.

d AUC: area under the curve.

e LiDAR: light detection and ranging.

f RFID: radio-frequency identification.

Table 3 summarizes the reported accuracy across the ML architecture categories included in this review. Mean reported accuracy ranged from 85.7% for YOLO-based approaches to 100% for the RFID-based approach. However, the latter was based on a single study with a small sample size. Similarly, the R-CNN–based and underwater computer vision categories each comprised only one study. Overall, the findings indicate that a range of ML approaches have achieved high reported accuracies, although direct comparisons should be interpreted with caution because the studies were conducted using different datasets and evaluation settings. The studies differed in ML architectures, sensing modalities (vision, sonar, RFID, and multimodal systems), datasets, evaluation protocols, and reported performance metrics. In addition, accuracy was the only performance metric reported consistently across a sufficient number of studies, whereas precision, recall, *F*_1_-score, and inference latency were reported inconsistently. Consequently, a descriptive synthesis was considered more appropriate than a statistical meta-analysis.

**Table 3.** Summary of reported accuracy by machine learning architecture.

Method category	Studies, n	Studies reporting accuracy, n	Accuracy range, %	Mean reported accuracy, %
YOLO^a^-based	7	4	72.2‐98.1	85.7
CNN^b^-based	5	3	85.6‐96	93.5
Pose-based	2	2	89.4‐95	92.2
Sonar-based	2	2	82‐99.4	90.7
Multimodal (vision+sensor)	2	2	85‐90	87.5
R-CNN–based	1	1	94.1	94.1
RFID^c^-based	1	1	100	100
Underwater computer vision (device-based)	1	0	Not reported	—^d^
Architecture not clearly specified	9	2	89.4‐99	94.2

a YOLO: You Only Look Once.

b CNN: convolutional neural network.

c RFID: radio-frequency identification.

d Not applicable.

## Discussion

### Overview of Key Findings

This review synthesizes current evidence on ML-based drowning detection and highlights both methodological progress and critical limitations. The findings show that the field is dominated by detection-based systems using supervised learning, with CNN and YOLO-based visual architectures being the most commonly adopted approaches [8,11,22]. Reported performance is generally high, particularly in terms of accuracy; however, evaluation practices are heavily skewed toward this single metric, with limited reporting of precision, recall, latency, and false alarm rates.

In addition, most systems are developed and evaluated using private datasets under controlled conditions, with minimal real-world validation and no evidence of external or cross-dataset testing [24,28]. Real-world challenges such as glare, occlusion, and crowding are rarely incorporated into evaluation frameworks, and multimodal approaches remain limited [25,33]. Together, these findings indicate that while methodological development is advancing, evidence of real-world reliability remains insufficient.

### Methodological Patterns and Evaluation Limitations

The dominance of detection-oriented, supervised learning systems suggests that the field has converged around a relatively narrow set of approaches. While these methods have demonstrated strong performance, this convergence may limit innovation and reduce the ability of systems to capture the complexity of drowning behavior, which is inherently subtle and temporally dynamic [13,49,50].

Only one of the 30 included studies evaluated vision-language models (VLMs), specifically contrastive language-image pretraining and bootstrapping language-image pretraining for swimmer-behavior question answering with light detection and ranging depth data, while the remaining studies used CNN- and YOLO-based architectures. This suggests that VLMs are still underrepresented in the current literature and warrant further investigation for real-time drowning detection.

At the same time, evaluation practices reveal a critical limitation. Accuracy remains the most frequently reported metric, despite drowning detection being a rare-event problem where false positives and false negatives have serious consequences [43]. Other key metrics, including precision, recall, *F*_1_-score, latency, and frame rate, are inconsistently reported. As a result, many systems demonstrate strong classification performance without providing sufficient evidence of operational effectiveness, particularly in terms of timely detection and false alarm control.

### Research-to-Deployment Gap and Real-World Robustness

A central finding of this review is the persistent gap between laboratory performance and real-world readiness. Although many studies claim real-time capability, only a very small number provide validation under real-world conditions, and none report external or cross-dataset evaluation. This discrepancy arises because real-time performance is typically assessed using inference speed (eg, frames per second or latency) on offline video datasets rather than end-to-end system performance, which includes alert generation, communication, and rescue response under operational conditions [13,33,45,49,51]. As a result, high inference speed alone does not guarantee effective real-world deployment. Similar challenges have been observed in other safety-critical domains, where staged validation frameworks have been adopted to bridge the gap between laboratory performance and operational use. For example, autonomous vehicle perception systems undergo progressive evaluation through simulation, controlled environments, and supervised real-world testing before deployment, while AI-based medical devices require prospective real-world validation before regulatory approval. In contrast, drowning detection research lacks an equivalent validation framework. Establishing standardized deployment-oriented evaluation protocols, such as supervised pilot studies with lifeguard oversight, would improve the translation of laboratory findings into reliable real-world systems.

This gap is further reinforced by the limited consideration of real-world challenges in model development and testing. Key disturbances such as glare, occlusion, crowding, and viewpoint variation are rarely addressed, despite being common in operational environments. Even when specific challenges are considered, they are often handled in isolation rather than as part of a comprehensive robustness framework.

In addition, the dominance of RGB and underwater vision systems reflects practical advantages but also highlights limitations in robustness, particularly when modalities are evaluated independently [24,28,33,34]. The limited adoption of multimodal approaches suggests that the field has not yet fully leveraged strategies that could improve detection across diverse and challenging conditions. One potential barrier is the lack of a standardized, publicly available multimodal benchmark dataset. Collecting synchronized RGB video, underwater imagery, sonar, and sensor data requires considerably more infrastructure and resources than single-camera studies, forcing individual research groups to develop their own datasets. Similar challenges were observed in autonomous driving before the release of synchronized multimodal benchmarks, such as the Waymo Open Dataset [52], which substantially accelerated multimodal perception research by providing shared, standardized datasets. Developing a comparable benchmark for drowning detection, even in a controlled swimming pool environment with synchronized RGB, underwater, and sensor data, could lower barriers to research, improve comparability across studies, and accelerate the development of robust multimodal detection systems.

### Reproducibility, Ethics, and Future Research Directions

The review also identifies significant challenges related to reproducibility and data transparency. All included studies relied on private datasets, and many did not clearly report dataset characteristics such as sample size or data type [28,49,51]. This lack of standardization limits comparability across studies and hinders independent validation, indicating that advances in model development have not been matched by progress in evaluation infrastructure.

Privacy and ethical considerations also remain underaddressed, despite their importance for real-world deployment. Only a minority of studies explicitly discussed privacy concerns, even though many systems rely on continuous visual monitoring in sensitive environments [25]. Nonvisual sensing approaches may offer more privacy-preserving alternatives and should be explored further.

Future research should prioritize the development of standardized evaluation frameworks, public benchmark datasets, and external validation protocols. In addition, greater emphasis is needed on comprehensive performance reporting, incorporation of real-world challenges, multimodal system design, and privacy-aware methodologies. Addressing these gaps will be essential for translating current advances into reliable and deployable drowning detection systems. Beyond the single VLM-based study identified, general-purpose VLMs such as Gemini, MiniCPM-V, and the Qwen-VL series remain largely unexplored for drowning detection. Given their strong zero- and few-shot capabilities, they represent a promising direction for future research. However, their suitability for real-time, safety-critical deployment remains unverified due to their high computational demands and the lack of benchmarking on inference latency and false alarm rates.

### Conclusion

This review demonstrates that while ML has achieved remarkable progress in drowning detection, current systems remain largely experimental and fall short of real-world reliability. The widespread reliance on curated datasets, limited external validation, and inconsistent evaluation practices exposes a critical gap between reported performance and practical deployment. Bridging this gap demands a shift toward rigorous validation, standardized evaluation frameworks, and robust, privacy-aware, real-time systems. Advancing in this direction is not merely a technical necessity but a public health imperative, essential for translating promising innovations into scalable, life-saving drowning prevention solutions.

## Supplementary material

10.2196/100939 Multimedia Appendix 1 Databases and search terms used in the literature search.

10.2196/100939 Multimedia Appendix 2 PICO (Problem, Intervention, Comparison, Outcome) framework of the research questions.

10.2196/100939 Multimedia Appendix 3 PROBAST: assessment of the risk of bias for each study.

10.2196/100939 Checklist 1 PRISMA checklist.

10.2196/100939 Checklist 2 TRIPOD Checklist

## Abbreviations

CLAIM: Checklist for Artificial Intelligence in Medical Imaging
CNN: convolutional neural network
ML: machine learning
PICO: Problem, Intervention, Comparison, and Outcome
PRISMA: Preferred Reporting Items for Systematic Reviews and Meta-Analyses
PROBAST: Prediction Model Risk of Bias Assessment Tool
QUADAS-2: Quality Assessment of Diagnostic Accuracy Studies 2
R-CNN: region-based convolutional neural network
RFID: radio-frequency identification
RGB: red, green, and blue
RQ: review question
TRIPOD: Transparent Reporting of a Multivariable Prediction Model for Individual Prognosis or Diagnosis
VLM: vision-language model
YOLO: You Only Look Once
