# Supplementary material for: Advances in Machine Learning Paradigms for Reliable Swimming Pool Drowning Detection: Systematic Review of Methods and Implementations
Source: JMIR Rehabil Assist Technol. 2026 Nov 3;13:e100939. doi: 10.2196/100939 (PMC13730513; doi:10.2196/100939)
Supplement: Supplementary file 2 [file rehab-v13-e100939-s001.docx]

**Multimedia Appendix 1: Databases and Search Terms Utilized in Literature Search**

A comprehensive search strategy was developed and applied across six major electronic databases: **IEEE Xplore, Scopus, Web of Science, ACM Digital Library, PubMed, and Google Scholar**. The search strings were tailored to each database, incorporating Boolean operators, indexing features, and controlled vocabulary where applicable. The search strings were as follows:

| **Database** | **Search Terms** | **Records Retrieved** |
| --- | --- | --- |
| IEEE Xplore | (“drowning detection” OR “swimmer distress” OR “aquatic safety” OR “near drowning”) AND (“machine learning” OR “deep learning” OR “computer vision” OR “YOLO” OR “CNN” OR “LSTM”) AND (“detection” OR “classification” OR “real-time” OR “monitoring”) | 412 |
| Scopus | (“drowning detection” OR “swimmer distress” OR “aquatic safety”) AND (“machine learning” OR “deep learning” OR “computer vision”) AND (“classification” OR “real-time monitoring”) | 1,125 |
| Web of Science Core Collection | (“drowning detection” OR “swimming pool safety” OR “distress detection”) AND (“machine learning” OR “deep learning” OR “computer vision”) AND (“classification” OR “monitoring”) | 978 |
| ACM Digital Library | (“drowning detection” OR “aquatic safety” OR “swimmer monitoring”) AND (“machine learning” OR “deep learning” OR “computer vision”) AND (“real-time detection” OR “classification”) | 356 |
| PubMed | (“drowning” OR “near drowning” OR “water safety”) AND (“machine learning” OR “artificial intelligence”) AND (“detection” OR “monitoring”) | 214 |
| Google Scholar | (“drowning detection” OR “swimmer distress detection”) AND (“machine learning” OR “deep learning” OR “computer vision”) AND (“real-time” OR “classification” OR “monitoring”) | 1,842 |
| **Total** |  | **4,927** |
