# Supplementary material for: Advances in Machine Learning Paradigms for Reliable Swimming Pool Drowning Detection: Systematic Review of Methods and Implementations
Source: JMIR Rehabil Assist Technol. 2026 Nov 3;13:e100939. doi: 10.2196/100939 (PMC13730513; doi:10.2196/100939)
Supplement: Supplementary file 3 [file rehab-v13-e100939-s002.docx]

**Multimedia Appendix 2: Problem, Intervention, Comparison, Outcome (PICO) Framework of Research Questions**

| **PICO** | **Concepts** | **Related Terms for Search** |
| --- | --- | --- |
| **Problem** | Drowning Detection and Aquatic Safety | "drowning detection," "drowning recognition," "swimmer distress," "water safety," "aquatic monitoring," "swimming pool surveillance," "near-drowning," "submersion detection," "water accident detection," "human activity in water" |
|  | Environmental and Operational Challenges | "occlusion," "glare," "water reflection," "lighting variation," "multiple swimmers," "crowding," "viewpoint variation," "underwater distortion," "surface turbulence," "real-time monitoring," "rare event detection" |
| **Intervention** | Machine Learning and Computer Vision Techniques | "machine learning," "deep learning," "artificial intelligence," "computer vision," "neural networks," "CNN," "RNN," "LSTM," "transformer," "YOLO," "object detection," "human activity recognition," "anomaly detection" |
|  | Spatio-Temporal Modeling and Detection Frameworks | "spatio-temporal models," "3D CNN," "video analysis," "motion detection," "behavior recognition," "pose estimation," "optical flow," "attention mechanisms," "sequence modeling" |
| **Comparison** | Model Architectures and Learning Paradigms | "CNN vs transformer," "YOLO vs traditional methods," "supervised learning," "unsupervised learning," "semi-supervised learning," "rule-based vs machine learning," "single modality vs multimodal" |
|  | Validation and Evaluation Strategies | "internal validation," "external validation," "cross-dataset evaluation," "real-world testing," "simulation," "benchmark comparison," "generalization," "robustness evaluation" |
| **Outcome** | Detection Performance Metrics | "accuracy," "precision," "recall," "F1-score," "sensitivity," "specificity," "AUC," "false alarm rate," "false positive rate," "missed detection rate" |
|  | Real-Time Performance and Deployment | "latency," "real-time detection," "frame rate," "FPS," "response time," "computational efficiency," "edge deployment," "embedded systems" |
|  | Generalizability and Practical Impact | "generalizability," "robustness," "real-world applicability," "deployment readiness," "scalability," "operational reliability," "public health impact," "drowning prevention" |
