# Supplementary material for: Advances in Machine Learning Paradigms for Reliable Swimming Pool Drowning Detection: Systematic Review of Methods and Implementations
Source: JMIR Rehabil Assist Technol. 2026 Nov 3;13:e100939. doi: 10.2196/100939 (PMC13730513; doi:10.2196/100939)
Supplement: Supplementary file 4 [file rehab-v13-e100939-s003.docx]

**Multimedia Appendix 3: Assessment of Risk Bias for Each Study**

| Title | Authors | Data Source Bias | prediction Bias | Outcome Assessment Bias | Analysis Bias | Overall Risk |
| --- | --- | --- | --- | --- | --- | --- |
| SwimTrack: Drowning Detection using RFID | Farzan Dehbashi et al. | High | Low | Moderate | Moderate | High |
| Intelligent Monitoring Drowning Early Warning System | Wei Wang et al. | High | Moderate | Moderate | High | High |
| Comparative Analysis of Underwater Drowning Detection Using Convolutional Neural Networks and Transfer Learning | Arsanchai Sukkuea et al. | Low | Low | Moderate | Low | High |
| A Drowning Detection Model by Integrating Multi-Scale Enhanced Receptive Fields with the YOLOv8 Framework | Lin Fan et al. | High | Low | Moderate | Moderate | High |
| A Novel Drowning Detection System and Its Performance Analysis | Thannicha Iampanit et al. | High | Low | Moderate | Moderate | High |
| A Water Behavior Dataset for an Image-Based Drowning Solution | Saifeldin Hasan et al. | Moderate | Low | Moderate | Moderate | High |
| An Improved Swimming Pool Drowning Detection Method Based on YOLOv8 | Tianyi He et al. | High | Low | Moderate | Low | High |
| Application of Deep Learning for the Classification of Drowning Incidents | Türkalp Türker Ünlü et al. | Moderate | Low | Moderate | Low | High |
| Automated Wearable Device to Detect Drowning Incidents in Water Bodies | Mustafa Basthikodi et al. | Low | Low | High | Low | High |
| Computer Vision Enabled Drowning Detection System | Upulie Handalage et al. | Moderate | Low | Moderate | High | High |
| Cygnus: A Vision-Based Drone System for Drowning Detection Using IoT | Dhumravarna Ambre et al. | Moderate | Low | Moderate | Low | High |
| An Improved YOLOv5 Algorithm for Drowning Detection in the Indoor Swimming Pool | Ruiliang Yang; Kaikai Wang; Libin Yang | Moderate | Low | Moderate | Low | High |
| MS-YOLO: A Lightweight and High-Precision YOLO Model for Drowning Detection | Qi Song et al. | Moderate | Low | Moderate | Low | High |
| Drowning Detection Based on YOLOv8-Pose and Human Pose Estimation | Luyao Chang et al. | Moderate | Low | Moderate | Low | High |
| A Video Drowning Detection Device Based on Underwater Computer Vision | Tingzhuang Liu et al. | High | Low | Moderate | High | High |
| DrownNet: Infant Drowning Alert System Using Deep Learning | Lipika M. S. et al. | High | Low | Moderate | Moderate | High |
| A Pool Drowning Detection Model Based on Improved YOLO | Wenhui Zhang; Lu Chen; Jianchun Shi | High | Low | Moderate | Low | High |
| YPMNet: A Lightweight Drowning Detection Algorithm | Xigui Lei et al. | High | Low | Moderate | Moderate | High |
| Improved YOLOv7 Fusion Detection Line for Swimming Pool Drowning Detection | Bing Bai et al. | High | Low | Moderate | Low | High |
| Mask R-CNN Based Real-Time Near-Drowning Person Detection System in Swimming Pools | Muhammad Aftab Hayat et al. | Moderate | Low | Moderate | Moderate | High |
| Construction Method of Swimming Pool Intelligent Assisted Drowning Detection Model Based on Computer Feature Pyramid Networks | Keshi Li et al. | Moderate | Low | Moderate | High | High |
| Development of Neural Network Algorithms for Early Detection of Drowning in Swimming Pools | Roman Vestnikov et al. | High | Low | Moderate | High | High |
| Enhancing Water Safety in the Philippines: Early Detection of Drowning Patterns through Wearable Devices and Machine Learning Algorithms | Gadiel Nathan C. Arriesgado et al. | High | Low | Moderate | High | High |
| Development of a Bathing Accident Monitoring System Using a Depth Sensor | Yoshiaki Endo et al. | Moderate | Low | Moderate | Low | High |
| Deep Learning Used to Recognition Swimmers Drowning | Jia-Xian Jian et al. | High | Low | Moderate | High | High |
| To Detect Active Drowning Using Deep Learning Algorithms | Belshia Jebamalar G et al. | High | Moderate | Moderate | High | High |
| SwimVQA: A Multimodal Approach to Drowning Detection by Fusing VQA and LiDAR | Muhammad Zeeshan Khan et al. | High | Low | Moderate | High | High |
| Automated and Intelligent System for Monitoring Swimming Pool Safety Based on the IoT and Transfer Learning | Aziz Alotaibi et al. | Low | Low | Low | Moderate | Moderate |
| Proposal of a Swimming Pool Drowning Detection System Using Cameras and Raspberry Pi Based on Machine Learning | Christopher Urruchi et al. | High | Low | Moderate | Low | High |
